# Inappropriate antibiotic prescribing for acute bronchiolitis in Colombia: a predictive model

**DOI:** 10.1186/s40545-020-00284-6

**Published:** 2021-01-04

**Authors:** Jefferson Antonio Buendía, John Edwin Feliciano-Alfonso

**Affiliations:** 1 Grupo de Investigación en Farmacología Y Toxicología, Departamento de Farmacología Y Toxicología, Facultad de Medicina, Universidad de Antioquia, 51D #62-29 Medellín, Colombia; 2 Departamento de Medicina Interna, Facultad de Medicina, Universidad Nacional de Colombia, Bogotá, Colombia

**Keywords:** Acute bronchiolitis, Antibiotics, Colombia

## Abstract

**Introduction:**

Acute bronchiolitis is the leading cause of hospitalization in the pediatric population. The inappropriate prescription of antibiotics in acute bronchiolitis is associated with bacterial resistance, higher costs, and risk of adverse effects in this population. The objective of this work is to develop a predictive model of inappropriate use of antibiotics in children with acute bronchiolitis in Colombia.

**Methods:**

A retrospective cohort study was conducted in patients under 2 years of age with a diagnosis of acute bronchiolitis from two hospitals in Rionegro, Colombia. To identify factors independently associated with inappropriate use of antibiotics, we used logistic regression and estimated odds ratios (ORs). To assess discrimination, area under the curve (AUC) was estimated with a 95% confidence interval and plotted using AUC–ROC plots. To correct sampling bias of variance parameters and to evaluate the internal validity of the model, repeated curved validation “tenfold cross-validation” was used, comparing the area under the ROC curve obtained in the repetitions with that observed in the model

**Results:**

A total of 415 patients were included. 142 patients (34.13%) had a prescription of some antibiotic during their hospital stay. In 92 patients (64.78%, 95% CI 56.3 to 72.6%) the prescription of antibiotics was classified as inappropriate. Age older than 1 year, chest retractions, temperature between 37.5 °C and 38.5 °C and leukocyte count between 10,000 and 15,000 million/mm^3^ were the predictive variables of inappropriate use of medications in this population.

**Conclusion:**

The presence of fever between 37.5 °C and 38.5 °C, leukocytosis between 10,000 and 15,000 million/mm^3^, and age older than 1 year and presence of chest retractions, should alert the physician regarding the high risk of inappropriate prescription of antibiotics. Patients with acute bronchiolitis with a score on our scale greater than 2 should be carefully evaluated regarding the need for the use of antibiotics, if prescribed.

## Introduction

Acute bronchiolitis is the main cause of hospitalization in the pediatric population [1]. This disease constitutes a constant challenge due to its frequency and morbidity, as well as its wide diagnostic and therapeutic variability [2]. This variability is associated with high costs and poor health outcomes [3]. In developed countries, this disease is related to high direct and indirect costs for both the health system and families [4–6]. In this regard, it is necessary to continue researching strategies that allow the early detection of such inappropriate practices.

Predictive models in acute bronchiolitis have been characterized by their low diagnostic validity and selection biases as they are usually validated in a high-risk or hospitalized population [7]. The inappropriate prescription of antibiotics in viral respiratory infections such as acute bronchiolitis is associated with bacterial resistance, high costs and risk of adverse effects [8]. In the United States, during the years 2007 to 2015, about 1 in 4 patients with acute bronchiolitis received an antibiotic during their emergency care [9]. Few models have been published about the risk of inappropriate antibiotic prescription [7]. The objective of this research is to create a predictive model of inappropriate use of antibiotics in children with acute bronchiolitis in Colombia. This instrument is expected to contribute to the adequate use of resources in the pediatric population.

## Methods

This is a retrospective cohort study in patients under 2 years of age admitted to the emergency department with a diagnosis of acute bronchiolitis (Code ICD-10: J21.0, according to the definition of the Colombian guideline for acute bronchiolitis) [10] in two hospitals located in Rionegro, Antioquia, from January 1, 2018 to December 31, 2018. This municipality is located in the department of Antioquia, it has around 101,046 inhabitants and is the main urban center in the east of this department [11]. We included patients under 2 years of age admitted to the emergency room with a diagnosis of acute bronchiolitis by the pediatrician. Patients with a confirmed diagnosis of whooping cough or with positive bacterial cultures at the time of admission that suggested an alternative diagnosis of bacterial infection on admission were excluded. The study was approved by the ethics committees of Universidad de Antioquia (No. 18/2015) and Clínica Somer (No. 281015).

### Study variables

The electronic medical records of the included patients were reviewed and information was extracted regarding sex, weight, age, signs and symptoms on admission to hospital. Pathological antecedents such as prematurity, bronchopulmonary dysplasia, chronic or neurological cardiopulmonary disease or other comorbidities were also recorded. Likewise, information was extracted regarding the in-hospital evaluation, use of medications, and acute complications. Inappropriate antibiotic use was defined, in accordance with previous studies [8, 9], as: (i) patients with an antibiotic prescription during hospitalization and with the absence of confirmed bacterial infection (blood culture, urine culture, cerebrospinal fluid culture, chest X-ray with alveolar consolidation, etc.); (ii) patients without suspected sepsis documented in the medical history; (iii) patients without a diagnosis of acute otitis media or other infection in bone or soft tissues documented in the medical history.

### Sample size

Estimates were made according to Riley’s recommendations for estimating sample sizes for multivariate predictive models with a binary outcome [12]. Assuming 6 potential predictor variables, IAU prevalence of 50% [9], the proportion of unexplained variation of 0.15 (as recommended by Riley in the absence of previous good-quality information), shrinkage of 0.9, marginal error in the estimation of the intercept of 0.05, and an acceptable difference between the apparent and adjusted *R*^2^ (Cox–Snell *R*-squared statistic) of 0.05, the sample size required for the development of this new model was 385 patients.

### Statistical analysis

Continuous variables were shown as mean ± standard deviation (SD) or median (interquartile range [IQR]), whichever was appropriate. Categorical variables were shown as numbers (percentage). To identify factors independently associated with inappropriate use of antibiotics, we used logistic regression and estimated odds ratios (ORs) and adjusted for potential confounding variables.

Initially, based on univariate logistic regression, we included only in the final logistic regression model variables associated with inappropriate use of antibiotics with values of *p* < 0.2 or that changed the effect estimate by over 10% after their inclusion. A multivariate logistic regression model was performed with a backward elimination method, used with a *p* value of 0.05 as the limit value for the model entry. The variable selection and modeling processes were made following the recommendations of Greenland [13]. The method of Sullivan et al. was used to generate the risk scores and estimate the risks observed with each score [14]. To assess discrimination, area under the curve (AUC) was calculated with a 95% confidence interval and plotted using AUC–ROC plots [15]. To correct prediction probabilities for over-optimistic predictions and to evaluate the calibration, the model was analyzed by comparing the predicted probability to the observed probability of inappropriate antibiotic use and examined with a calibration plot and calibration slope with 95% CI. Calibration plots (STATA function: pmcalplot) displayed observed risk by deciles of the predicted risk and examined risk at the individual level using Locally Weighted Scatterplot Smoothing algorithms [16]. We also calculated the Hosmer–Lemeshow goodness-of-fit test as well as calibration curves between the predicted probabilities and the observed data. To correct sampling bias of variance parameters and to evaluate the internal validity of the model, repeated curved validation “tenfold cross-validation” was used, comparing the area under the ROC curve obtained in the repetitions with that observed in the model [15, 16]; as well as with the bootstrapping technique with which both the adjusted oppressiveness and area values were estimated. All statistical tests were two-tailed, and the significance level used was *p* < 0.05. The data were analyzed with Statistical Package Stata 15.0 (Stata Corporation, College Station, TX).

## Results

### Characteristics of the study population

A total of 415 patients were included. The sociodemographic and clinical characteristics of the population are shown in Table 1. The studied population had a median hospital stay of 3.69 days (interquartile range of 4.06 days). 80 patients (19.23%) developed bacterial pneumonia, 19 patients (4.57%) had sepsis, 8 patients (1.92%) developed atelectasis and only 1 patient had tension pneumothorax. Furthermore, 56 patients (13.46%) were referred to the intensive care unit, and there were no deaths during the study period.

**Table 1:** Characteristics of population

	*n* (%)
Age ≤ 12 months	345 (83.1)
Male sex	251 (60.3)
Prematurity	81 (19.4)
Comorbidities (CHD or neurological)	20 (4.8)
BPD	17 (4.1)
Atopy	17 (4.1)
Poor feeding	242 (58.1)
Exposure to cigarette smoking	49 (11.7)
Exclusive maternal breastfeeding for at least 6 months	102 (24.5)
RSV isolation	200 (48.1)
Dehydration	15 (3.6)
Nasal flaring and/or grunting	48 (11.5)
Chest retractions	184 (44.2)
Respiratory rate > 60	48 (13.3)
Oxygen saturation < 90%	238 (57.2)
Temperature (37.5 °C–38.5 °C)	119 (28.6)
Leukocytosis (10,000 a 15,000 million/mm^3^)	51 (13.0)

*BPD* bronchopulmonary dysplasia

### Use of antibiotics in the study population

142 patients (34.13%) were prescribed an antibiotic during their hospital stay. 84 patients (20.19%) were prescribed beta-lactams, 18 patients (4.33%) macrolides, 4 patients (0.96%) aminoglycosides, and 36 patients (8.65%) combined antibiotic therapy. In 92 patients (64.78%, 95% CI 56.3 to 72.6%) the prescription of antibiotics was classified as inappropriate. Within the antibiotic subgroups, 76%, 83%, and 36% of beta-lactams, macrolides, and combined therapy were classified as inappropriate, respectively. In contrast, none of the aminoglycosides was classified as inappropriate (all four were administered in patients with proven urinary infection).

### Results of the multivariate regression analysis

**Fig. 1:**
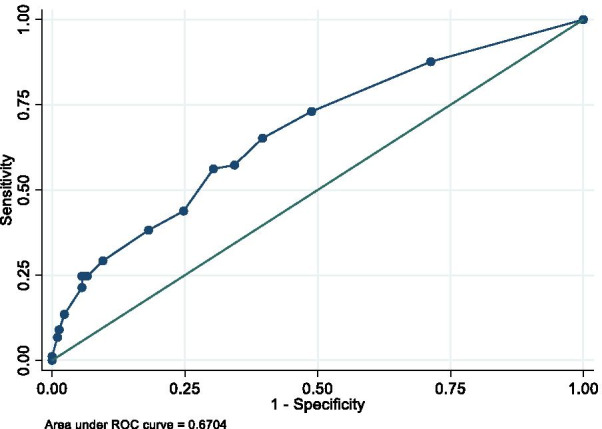
ROC curve

After entering all those variables with a value of *p* < 0.2 to the model (age, presence of intercostal retractions, temperature between 37.5 °C and 38.5 °C and leukocyte count between 10,000 to 15,000 million/mm^3^, prematurity, comorbidities, dehydration, oxygen saturation < 90%, and exposure to cigarette smoking) and performing the selection and modeling as described in the “Methods” section, the variables with which not only statistical significance was maintained, but also achieved the highest final adjustment were age, presence of intercostal retractions, temperature between 37.5 °C and 38.5 °C and leukocyte count between 10,000 to 15,000 million/mm^3^ (see Table 2). After calculation, the sensitivity, specificity, positive and negative predictive values were 90%, 98.6%, 66.67%, 78.6%, with a percentage of correctly classified cases of 78.26%. The scores established for each of these variables within the predictive scale and the calculation of the risk of inappropriate use of antibiotics with these scores can be seen in Tables 2 and 3. The risk score model had an excellent calibration and goodness of fit through the Hosmer–Lemeshow test (*p* = 0.41). The tenfold cross-validation revealed a corrected average (AUC 0.67 IC 95% 0.67–0.68) value very close to the acceptability limit of 0.7 (see Fig. 1), with mean over-optimism obtained by a bootstrapping of 0.65 (min 0.54, max 0.74).

**Table 2:** Selected predictor variables for multivariable model

Variable	OR (IC 95%)	Points
Chest retractions		
No	Reference	0
Yes	1.67 (1.02–2.75)	1
Temperature (37.5 °C–38.5 °C)		
No	Reference	0
Yes	2.10 (1.24–3.54)	1
Leukocytosis (10,000 a 15,,000 million/mm^3^)		
No	Reference	0
Yes	2.58 (1.36–4.91)	2
Age		
0–12 months	Reference	0
12–24 months	2.08 (1.11–3.90)	1

**Table 3:** Risk levels

Point total	Estimate of risk
0	29.18%
1	52.83%
2	75.27%
3	89.22%
4	95.74%
5	98.39%

## Discussion

These findings enabled a scale to be proposed, which is composed of 4 clinical variables that allow the prediction of the risk of inappropriate prescription of antibiotics in hospitalized patients with acute bronchiolitis with high specificity. This tool can classify patients according to their risk and, in this way, preventive actions can be taken. For example, it is possible to have a more parsimonious and careful behavior in the prescription of antibiotics in these groups. On this scale, the variable with the highest weight and score was the age of the patient. Patients between the ages of 12 to 24 months have a higher risk of inappropriate antibiotic prescription. This finding is consistent with previous literature reports. Papenburg et al. after analyzing records of 612 children with acute bronchiolitis in the United States from 2007 to 2015, found a frequency of inappropriate use of antibiotics (IUA) use of 70%. When evaluating variables associated with IUA, a higher risk (OR 2.64 95% CI 1.06–6.59) of inappropriate use of antibiotics was found in children between 12 and 24 months compared to children under 1 year.

One study found a higher risk (OR 2.64 95% CI 1.06–6.59) of inappropriate use of antibiotics in children between 12 and 24 months compared to children under 1 year. Poole et al. in 29 million visits of children to emergency services, found that 32% of antibiotics did not have a correct indication, with the highest percentages of antibiotic prescription (29%) in patients older than 1 year and younger 5 years old, and also in children under 1 year (22%) [17]. Zhang et al. found a 30% higher adjusted risk of inappropriate antibiotic prescription in those older than 6 years compared to those younger than 6 years after analyzing records of 8155 patients in China [18]. It is possible that doctors are much more careful with the prescription of antibiotics in children under one year due to the higher frequency of viral infections compared to bacterial infections, which perhaps leads them to have more “liberal” behaviors in older patients of 1 year and thus lead to a higher frequency of inappropriate antibiotic prescription.

Fever has also been documented as more frequent in patients with inappropriate use in previous studies. Patra et al. in an observational study of 47 infants, reveals not only a higher frequency of fever in patients who received antibiotics compared to those who did not receive it (65% vs. 38%, respectively), but also persistence of this symptom after 48 h in the group that received an antibiotic, suggesting its inappropriate indication in these patients [19]. Similarly, the study by Ababneh et al. which included 3883 pediatric outpatients due to acute respiratory infection, found that 69.2% of patients had an inappropriate prescription of antibiotics, and fever increased the probability twice (OR 2.14 95% CI 1.78 to 2.49) compared to patients without fever [20]. Our model identified that patients with temperatures classified as mild fever between 37 °C and 38 °C are those who have the highest risk of inappropriate antibiotic prescription. As in our case, signs of respiratory distress have been markers of possible inappropriate use of antibiotics in other studies. In the study carried out by Patra et al. the presence of respiratory distress was much more frequent in patients with antibiotics compared to those without antibiotics (86% vs. 56%, *p* = 0.009) [19]. In our population, patients with chest retractions had a 67% higher risk of IUA, being a symptom that adds to the other variables with more weight such as age to confer risk. Thus, it is clear that requesting a hemogram in a patient generates more noise in the therapeutic approach than some type of benefit. In the literature, it is clear that if some tests should be requested in these patients, it is the isolation of RSV, since it has been shown that the latter does generate a therapeutic impact by reducing the inappropriate prescription of antibiotics [21].

Our study has limitations. First, it was based on a retrospective review of medical records, so information biases cannot be ruled out. However, the information on all the covariates included in the model was complete for all patients. Second, our study took data from the two main health centers in a municipality, limiting the generalizability of the results to patients seen at less complicated levels of care. However, the frequency of complications or history of comorbidity in the population or the level of severity on admission is not very different from those published from studies in less complicated care centers [22]. Extrapolation of the results to these scenarios should be taken with caution.

## Conclusion

The presence of fever between 37.5 °C and 38.5 °C, leukocytosis between 10,000 and 15,000 million/mm^3^, and age older than 1 year, and presence of chest retractions should alert the physician regarding the high risk of inappropriate prescription of antibiotics. Patients with acute bronchiolitis with a score on our scale greater than 2 should be carefully evaluated regarding the need for the use of antibiotics, if prescribed.

## Data Availability

The raw data supporting our findings can be requested at http://ciemto.medicinaudea.co/.

## Abbreviations

RSV: Respiratory syncytial virus
IUA: Inappropriate use of antibiotics
